# Cones of Pumice Stone

**Published:** 1870-11

**Authors:** A. Berry


### CONES OF PUMICE STONE.

BY A. BERRY.

When a Dentist makes a discovery or invention that will facilitate or expedite any operation of our specialty, it is desirable he should communicate it to the profession. If the advantage gained is small, still it is valuable, as time saved is important. On this account the following is offered:

After using most of the varieties of cones of cork, rubber, leather, soft wood, felt, etc., for polishing rubber and other plates, I was not satisfied with any of them. No one would cut rapidly enough.

Resolving to try cones of pumice stone, I made some and found them to answer admirably, as after using the steel bur on rubber they cut rapidly if kept wet, and if skillfully worked they produce a pretty smooth surface. Following this with a cork or leather cone and pulverized pumice, and then a wood cone (as making the surface smoother than the others), and the brush with whiting, gives a plate a finer finish and in much less time than is possible in the usual manner without the pumice cone.

This cone is easily made by sawing from the firm stone, in the direction of the grain, pieces an inch or more in diameter and two or more inches in length. Make a tapering hole in one end, into which fit nicely and retain by liquid silex or other cement a piece of wood by which to attach it to the lathe, and cut away the angles, making it a little tapering; secure it to the lathe, and keeping it wet, hold firmly against it a good sized piece of the stone, on which revolve it rapidly and it will soon become round and fit for use.

